# Plasticity of hippocampal memories in humans

**DOI:** 10.1016/j.conb.2017.02.004

**Published:** 2017-04

**Authors:** Aidan J Horner, Christian F Doeller

**Affiliations:** 1 Department of Psychology, University of York, York YO10 5DD, UK; 2 Kavli Institute for Systems Neuroscience, Centre for Neural Computation, The Egil and Pauline Braathen and Fred Kavli Centre for Cortical Microcircuits, NTNU, Norwegian University of Science and Technology, St. Olavs Hospital, Trondheim University Hospital, Trondheim, Norway; 3 Donders Institute for Brain, Cognition and Behaviour, Radboud University, Nijmegen, the Netherlands

## Abstract

The human hippocampus is a brain region that supports episodic and spatial memory. Recent experiments have drawn on animal research and computational modelling to reveal how the unique computations and representations of the hippocampus support episodic and spatial memory. Invasive electrophysiological recordings and non-invasive functional brain imaging have provided evidence for the rapid formation of hippocampal representations, as well as the ability of the hippocampus to both pattern-separate and pattern-complete input from the neocortex. Further, recent evidence has shown that hippocampal representations are in constant flux, undergoing a continual process of strengthening, weakening and altering. This research offers a glimpse into the highly plastic and flexible nature of the human hippocampal system in relation to episodic memory.

## Highlights

• Hippocampal neurons can rapidly associate to form event engrams.
• Pattern separation at encoding decreases interference between engrams.
• Pattern completion allows for the retrieval of an entire event.
• The hippocampus is constantly encoding and altering engrams.
• The plasticity and malleability of engrams is critical to supporting future decisions.

**Current Opinion in Neurobiology** 2017, **43**:102–109

This review comes from a themed issue on **Neurobiology of learning and plasticity**

Edited by **Leslie Griffith** and **Tim Vogels**

For a complete overview see the Issue and the Editorial

Available online 4th March 2017

**http://dx.doi.org/10.1016/j.conb.2017.02.004**

0959-4388/© 2017 The Authors. Published by Elsevier Ltd. This is an open access article under the CC BY license (http://creativecommons.org/licenses/by/4.0/).

## Introduction

Patients with lesions to the hippocampus have marked deficits in episodic [1] and spatial [2] memory. In particular, selective hippocampal damage, without damage to the surrounding medial temporal lobes (MTL), disrupts performance in tasks that test memory for multimodal associations [3, 4] and relational representations [5, 6]. Thus, the hippocampus is thought to support the *recollection* [7] of episodic events by representing the complex spatiotemporal patterns that uniquely define typical real-world events.

More recently, focus has shifted from the representations supported by the human hippocampus to the computations it performs. These were first postulated in the seminal work of Marr [8^•^], and have been further developed in recent decades [9, 10, 11]. This computational approach has been highly influential in the study of the rodent hippocampus [12, 13, 14], however it is only recently that it has informed research into the human hippocampus. The principle tenets of this approach are that the hippocampus is able to: (1) rapidly form associations between arbitrary stimuli—*one-shot learning*, (2) form distinct representations despite similar input from the neocortex—*pattern separation*, and (3) retrieve a complete representation in the presence of an ambiguous or partial input *—pattern completion*.

Despite the difficulties associated with studying learning-related plasticity in the human hippocampus, direct, invasive, electrophysiology as well as indirect, non-invasive, functional brain imaging allows us to infer the presence of these processes. Here we review recent electrophysiology and brain imaging studies in humans that reveal both the representational content and computations performed by the hippocampus. We focus on the three tenets of the computational model outlined above. Further, we discuss recent research that extends the role of the hippocampus, from encoding and retrieving distinct episodic memories, to modifying and integrating pre-existing memories into network-like mnemonic structures. This new avenue of research has highlighted the highly plastic and dynamic nature of the hippocampus. Ultimately, it is this flexibility that ensures our memories of the past are continually relevant to decision-making processes in the present.

## Rapid learning in the human hippocampus

**Figure 1:**
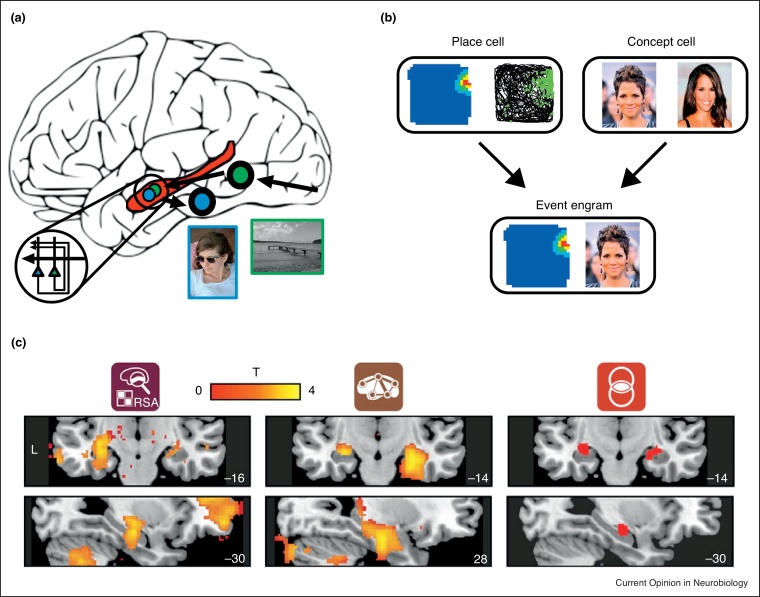
Event engrams in the human hippocampus. **(a)** Schematic of computational model of episodic memory. Distinct neocortical representations for event elements (*e.g.* locations and people) form links with individual neurons in the hippocampus (*e.g.* place and ‘concept’ cells). When experienced together, hippocampal place cells (green) and concept cells (blue) can rapidly form direct associations, forming ‘event engrams’. At retrieval, when the location is cued, the hippocampus receives a partial input. All associated elements are retrieved via the process of pattern completion, supported by the recurrent connections of subfield CA3 (simplified wiring diagram of CA3 in zoomed in panel in bottom left) and subsequently the retrieved elements are reinstated in the neocortex, allowing for the experience of ‘recollection’. **(b)** Simplified example of an ‘event engram’. Place cells (example shown from a rodent, showing firing in the top right corner of the environment, with permission from Ref. [66]) and concept cells (*e.g.* a neuron that fires when presented with any image of Halle Berry) may act as the ‘building blocks’ of episodic memory, the formation of an ‘event engram’ results from these cells forming direct associations when experienced together, such that the associated concept cell will fire when the place cell fires (and vice versa). Note, event engrams are likely to be much more complex in nature than simple pairwise associations, and may include multiple (*i.e.* >2) elements, with direct connections between the neurons coding for each constituent element. **(c)** Evidence for the ‘convergence zone’ hypothesis—multivariate and graph-theoretic network analyses suggest the hippocampus represents multimodal pairwise associations (left) and demonstrates ‘hub-like’ properties (middle) during episodic retrieval (conjunction shown on right; with permission from Ref. [16^••^]).

No two real-world events are identical; each one is uniquely defined by its complex spatiotemporal characteristics. The individual elements of an event, such as the location you are in or the person you are talking to, are thought to be represented in distinct neocortical regions. The hippocampus is thought to receive input from these neocortical regions, acting as a *convergence zone* [15], rapidly binding together this multimodal information into a coherent *event engram* (Figure 1a,b) [8^•^]—a population of interconnected hippocampal neurons that represent the constituent elements of a specific event. Combining connectivity and pattern similarity measures of fMRI, recent research suggests that the human hippocampus represents associations between multimodal stimuli, whilst simultaneously acting as a ‘hub’ within an extended cortical network, during memory retrieval (Figure 1c) [16^••^], providing experimental evidence for Marr’s proposal [8^•^].

What form do these hippocampal event engrams take and how rapidly are they established? Invasive electrophysiological recordings of single neurons in the human hippocampus of patients with epilepsy have demonstrated the presence of cells that fire in response to unique environmental features. In line with rodent research [17], single neurons fire in relation to specific locations in virtual reality (VR) environments [18]. The existence of *place cells* and other spatially modulated neurons in the human hippocampus [19, 20], alongside fMRI studies of spatial/scene processing [21, 22•, 23, 24•, 25], confirm the spatial nature of representations in the hippocampus and surrounding MTL.

Neurons with non-spatial firing patterns have also been shown, specifically in relation to well-known celebrities, famous buildings, and animals [26•, 27]. These so-called *concept cells* can respond to the identity of a person in a stimulus-invariant manner. For example, one neuron was shown to respond to both the written name, and a photo of, Halle Berry [26^•^]. Despite ongoing debate concerning how such neurons are best conceptualised [28, 29, 30], recent evidence suggests they could potentially represent long-term real world (perhaps not task-specific) associations [31]. As such, they may represent non-spatial environmental features in an analogous manner to spatially modulated place cells—that is, in a stimulus-invariant manner over relatively long times-scales. The presence of both spatial and non-spatial cells that code for specific elements (*e.g.* locations and people) in the real world appears to provide the ideal ‘building blocks’ [32] for event engrams (Figure 1b). In short, event engrams can be rapidly formed via direct associations between hippocampal neurons coding for the individual elements of any specific event.

Importantly, these neurons appear to tune their respective firing fields relatively quickly. Concept cells can respond to individual researchers that the patient only met on the day of testing [33], and recent rodent research has shown that place cell firing fields become tuned after only a single visit to that specific location [34^••^]. Thus, at the level of individual neurons, the hippocampus represents any possibly behaviourally relevant element in the environment, and these representations can be formed rapidly.

Events are more complex than single locations or individuals though. The hippocampus therefore needs to rapidly form direct associations between these neurons. For example, if you met Halle Berry at the Eiffel Tower, the hippocampus needs to form an association between the neurons coding for both of these elements from that single encounter. Recent electrophysiology has shown that individual neurons, which initially fire selectively to a famous person and landmark, change their firing properties after exposure to a composite image (*i.e.* an image of both the person and landmark) such that they subsequently fire to either image in isolation [35^••^]. Further, following an object-location encoding task in a VR environment, place cells associated with the location of a specific object were shown to fire during a free recall task when participants recalled that specific object [36]. These studies suggest the rapid formation of direct associations between neurons in the hippocampus, supporting the subsequent retrieval of an episodic-like memory.

Real word events are more complex in nature than the simple pairwise associations tested in the studies presented above, involving multiple elements that form complex configural representations. Nonetheless, the studies support the concept that the hippocampus acts as a convergence zone, rapidly forming associations between stimuli represented in distinct neocortical regions. However, one critical outstanding question is how such hippocampal neurons continue to differentiate between specific elements in the environment—once Halle Berry has been seen at the Eiffel Tower, how is the hippocampus able to independently represent Halle Berry and the Eiffel Tower (allowing them to be separately incorporated into future events), whilst simultaneously maintaining a configural representation of the two elements?

## Pattern separation and pattern completion in the human hippocampus

Pattern separation refers to the production of distinct (orthogonal) non-overlapping representations from similar overlapping input. It decreases interference at retrieval by minimising the representational overlap between two similar events at encoding. The dentate gyrus (DG), with its large number of neurons (relative to its principal input, the entorhinal cortex) and sparse coding, is thought to primarily support pattern separation in the hippocampus.

**Figure 2:**
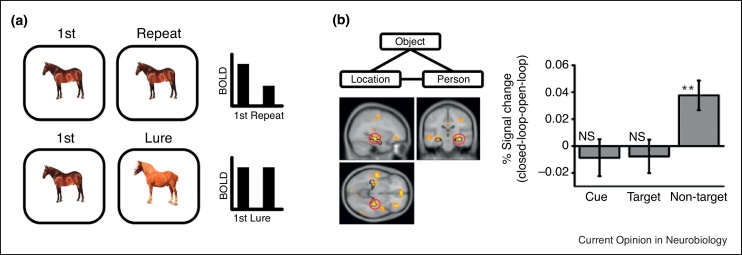
Pattern separation and pattern completion. **(a)** Evidence for pattern separation in the human hippocampus was first shown by Bakker *et al*. The repetition of a stimulus leads to the well-known phenomenon of adaptation, or repetition suppression. However, when a similar looking ‘lure’ image is shown, DG showed a BOLD response similar to the first presentation of the image (see Ref. [37^•^] for details). This suggests that DG is pattern separating the ‘lure’ image—encoding it as a separate representation despite the similar perceptual input. Example shown is illustrative, and does not present actual stimuli used or data presented. **(b)** Evidence for pattern completion in the human hippocampus has recently been shown by Horner *et al*. After learning location-object-person events (across three separate encoding trials, see Ref. [50^••^] for details), participants were tested on specific pairwise associations (*e.g.* cue location, retrieve object). Neocortical reinstatement was critically shown for the ‘non-target’ elements (*e.g.* person), suggesting all elements were retrieved and reinstated in the neocortex. Critically, the amount of reinstatement for ‘non-target’ event elements correlated with hippocampal BOLD response at retrieval, consistent with the proposal that the hippocampus retrieves all event elements via pattern completion, leading to their reinstatement in the neocortex (hippocampal image and % signal change graph with permission from Ref. [50^••^]).

fMRI has been used to provide evidence for pattern separation in the human hippocampus [37^•^]. The presence of pattern separation should mean that similar stimuli, for example two different images of an apple, are encoded as distinct representations. The authors used the well-known effect of adaptation, where repetition of the same stimulus results in reduced BOLD responses, to infer the presence of pattern separation. They took a release from adaptation when presented with a similar, but not identical, stimulus as a marker for pattern separation (Figure 2a). They saw this effect in a combined DG/CA3 region, but not in CA1 or surrounding MTL regions. Further studies have parametrically manipulated the similarity of repeated stimuli to show a non-linear mapping between the input and output of the hippocampus [38, 39], consistent with a pattern separation process. However, the studies presented pictures of objects rather than more complex events known to be supported by the hippocampus. Further, the results may be explicable in terms of a ‘match-mismatch’ signal unrelated to pattern separation [40].

More recently, videos of events with overlapping content have been combined with multivariate analyses of fMRI to provide further evidence of pattern separation [41]. By orthogonally combining two background contexts (scenes) with two foreground ‘events’ (people with objects), creating four related videos, the authors showed that representations in the hippocampus successfully distinguished each individual video. Further, multivariate analyses of high-resolution fMRI were used to successfully classify similar indoor scenes in DG (but not entorhinal cortex) [42]. Thus, despite the highly overlapping input, the hippocampus produced stable differentiated (*i.e.* pattern-separated) representations. Potential evidence has also been provided for a lack of pattern separation for similar VR environments (similar shops in different locations; however the effects may also reflect inappropriate pattern completion) [43]. Finally, recent research has shown the extent of pattern separation for related events (scene-face pairs) predicts subsequent memory interference [44], linking pattern separation at encoding with reduced interference at retrieval. The studies provide critical evidence for pattern separation, for simple objects and more complex episodic events and spatial environments, in the human hippocampus.

Pattern completion refers to the retrieval of a complete distinct representation (or ‘pattern’) given a partial or ambiguous input. It is thought to underlie our ability to recollect prior events from minimal cues. For example, we might see a picture of a friend and recollect all the details of a social occasion with them from the previous week. Hippocampal subregion CA3, with its dense recurrent connections, is thought to act as an attractor network [45, 46], underpinning pattern completion.

One way to assess pattern completion is to present participants with a single cue related to a complex event and assess whether all details of that event are subsequently retrieved. The presence of pattern completion predicts memory retrieval to be all-or-none, consistent with the ‘recollection’ component in dual process models of recognition memory [47]. Recent behavioural and fMRI evidence has been shown for this prediction. First, when complex events are learnt, for example location-person-object triplets, the retrieval success of elements within an event are related—if you retrieve one element correctly you are more likely to retrieve the other elements of the event successfully [48, 49•]. Using fMRI, it was shown that retrieval of these complex events was associated with a ‘reinstatement’ effect for all event elements in the neocortex, and that this reinstatement effect correlated with the hippocampal BOLD response [50^••^] (Figure 2b). Cuing with the location leads to reinstatement of the person and object, and this reinstatement correlates with hippocampal activity. This is consistent with a pattern completion process in the hippocampus driving reinstatement of the complete event. However, the reinstatement effect was specific to the category (*e.g.* people vs. locations), but not the element of the event (*e.g.* Barack Obama vs. Hilary Clinton), and therefore could not distinguish between reinstatement of the correct vs. incorrect within-category element. Further, the authors were unable to distinguish between different hippocampal subfields, so were not able to conclude the hippocampal signal originated specifically from CA3.

More recently, evidence for attractor dynamics in relation to spatial environments has been provided [51^•^]. Here, two distinct VR environments were learnt, and in a subsequent fMRI scanning phase, participants were placed in environments that morphed the surrounding landscape between the two learnt ‘endpoint’ environments. Consistent with rodent research [12], hippocampal representations for morphed environments showed a non-linear response where they became more similar to one of the endpoint representations across the trial, and this response predicted trial-by-trial mnemonic decision making. Thus, in the presence of an ambiguous cue, the hippocampus pattern completes to one of the learnt environmental representations.

In sum, fMRI has shown the presence of both pattern separation and pattern completion in the human hippocampus. What is unclear is the relationship between these two computations. Typically, pattern separation is thought to occur at encoding and pattern completion at retrieval, however the distinction between encoding and retrieval is not clear in the real world. How do these two processes, and the two hippocampal subfields (DG and CA3), interact to maximise our ability to not just recall a previous event accurately, but also to apply our memories of the past to guide future behaviour? One possibility is that the hippocampus temporally segregates, but rapidly alternates between, pattern separation and completion, consistent with models proposing the segregation of encoding and retrieval within separate phases of the hippocampal theta rhythm [52^•^].

This temporal segregation may also provide an answer to how a ‘Halle Berry’ and ‘Eiffel Tower’ neuron could maintain independent representations, whilst simultaneously maintaining a configural representation of the two elements. During specific phases of the theta rhythm, firing of hippocampal neurons will be primarily driven by neocortical input, and as such individual neurons will fire in relation to specific elements (*e.g.* Halle Berry). In other phases, firing will be driven by intra-hippocampal connections (*e.g.* recurrent connections in CA3), such that the ‘Halle Berry’ neuron will fire when presented with either Halle Berry or the Eiffel Tower.

## Post-encoding learning and plasticity

Our memories are as much about the future as the past. Ultimately, they must be behaviourally relevant to support decision-making processes. A new line of fMRI research has underlined how hippocampal representations are not static, but are malleable in nature—constantly being strengthened, weakened and altered in the presence of new information to ensure their continued behavioural relevance. The studies draw attention to the highly dynamic, plastic nature of representations in the hippocampus.

**Figure 3:**
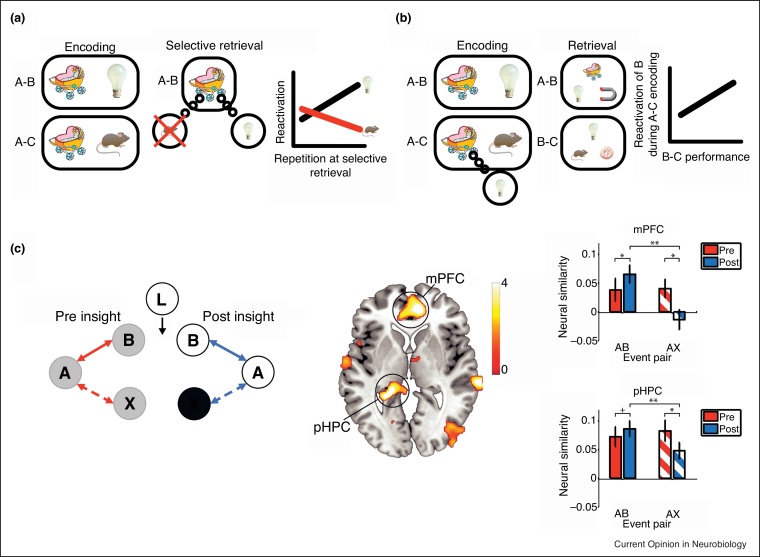
Suppression, generalization and integration of pre-existing memories. **(a)** Evidence for suppression of associated material in the neocortex during selective retrieval was first provided by Wimber *et al*. Participants learn A-B, then A-C pairs. They are then cued with A and asked to selectively retrieve B (not C). During selective retrieval, neocortical patterns associated with B increased, while those associated with C decreased, suggesting the suppression of the competing associated item. Critically, the extent of neocortical suppression predicted later forgetting, suggesting that retrieval can adaptively shape our episodic memories of the past. **(b)** Evidence for integration following reactivation was first provided by Zeithamova *et al*. Participants first learnt A-B pairwise associations, followed by A-C pairs. Memory for A-B and A-C pairs was tested, as well as ‘memory’ for the non-encoded pairs (B-C). The extent of reactivation (measured by pattern classification of fMRI data) of item B when learning the A-C pairs correlated with performance for the non-encoded pairs. This suggests reactivation at encoding can result in the formation of novel associations between items never seen together (the B-C pairs). Examples shown in (a) and (b) are illustrative, and do not present actual stimuli used or data presented (see Refs. [57,56^••^] for details). **(c)** Evidence that insight triggers the integration of separately learnt narrative structures in the hippocampus (and mPFC) was first shown by Milivojevic *et al*. Neural similarity (as measured with representational similarity analyses – RSA – of fMRI) between two separately learnt narratives (videos) increases after showing a ‘linking’ narrative (Section C with permission from Ref. [59^•^]).

First, in an awake delay period between learning and test, endogenous reactivation of specific event memories was seen in entorhinal and retrosplenial cortex [53^•^]. Importantly, the extent of reactivation correlated with subsequent memory performance. Thus, following initial encoding, memories are strengthened (or maintained) by a process of continuous reactivation in the MTL and neocortex. Interestingly, this endogenous reactivation also appears to facilitate learning of new overlapping material [54]. Memories can also be disrupted after encoding. When participants learn overlapping A-B and then A-C pairwise associations, the repeated retrieval of one pair (*e.g.* A-B) can result in poorer memory performance for the overlapping pair (*e.g.* A-C) [55]. Recent fMRI evidence shows that during retrieval of the A-B pair, the overlapping pair (A-C) is inhibited and the underlying representation is disrupted [56^••^] (Figure 3a). Thus, memories can be strengthened or weakened after initial encoding dependent on the extent of post-encoding reactivation or suppression.

Event memories can also be altered and integrated with existing memories in order to generalise to novel situations. After learning an A-B pairwise association, the reactivation of element B (measured with fMRI) when learning an overlapping A-C association correlates with participants’ subsequent performance on the non-encoded B-C pair [57] (Figure 3b). Further, the anterior hippocampus (and medial prefrontal cortex) appear to play a role in integrating representations for overlapping information [58^•^]. Thus, reactivation of previously learnt information can lead to generalisation between previously unseen elements. The hippocampus also appears to play a crucial role in the integration of separately learnt narrative structures when participants become aware that they relate to a larger coherent narrative, resulting in an insight-triggered reconfiguration of memory space [59^•^] (Figure 3c). Interestingly, these reconfigured narratives might be represented in a hierarchical manner along the long-axis of the hippocampus [60]. Related to this, the hippocampus also appears to represent the community structure of temporally related stimuli [61]. In sum, the representational space in the human hippocampus can be complex, structured and hierarchical, and most importantly, it is highly dynamic, constantly strengthening, weakening and altering existing representations to appropriately guide decision-making processes [62, 63••].

## Conclusion

Recent electrophysiology and functional brain imaging research has focused on the computations performed by the human hippocampus. In line with computational models, the human hippocampus appears to rapidly learn arbitrary associations between event elements, pattern separate overlapping neocortical input at encoding, and pattern complete partial neocortical input at retrieval. Thus, it is only recently that the mechanistic underpinnings of episodic and spatial memory in humans have been revealed. Further, research has suggested the hippocampus is involved in more than simply ‘remembering’. Rather, it supports a dynamic, plastic, flexible representational space that is continually altering and integrating memories of the past in order to guide decisions in the present [64, 65].

## Conflict of interest statement

Nothing declared.

## References and recommended reading

Papers of particular interest, published within the period of review, have been highlighted as:

• of special interest
•• of outstanding interest
